# Impact of Early-Life Microbiota on Immune System Development and Allergic Disorders

**DOI:** 10.3390/biomedicines13010121

**Published:** 2025-01-07

**Authors:** Norbert Dera, Katarzyna Kosińska-Kaczyńska, Natalia Żeber-Lubecka, Robert Brawura-Biskupski-Samaha, Diana Massalska, Iwona Szymusik, Kacper Dera, Michał Ciebiera

**Affiliations:** 1 Department of Obstetrics, Perinatology and Neonatology, Center of Postgraduate Medical Education, 01-809 Warsaw, Poland; nderrick@interia.pl (N.D.); katarzyna.kosinska-kaczynska@cmkp.edu.pl (K.K.-K.); robertsamaha@gmail.com (R.B.-B.-S.); iwona.szymusik@gmail.com (I.S.); 2 Warsaw Institute of Women’s Health, 00-189 Warsaw, Poland; diana_massalska@wp.pl (D.M.); michal.ciebiera@gmail.com (M.C.); 3 Department of Gastroenterology, Hepatology and Clinical Oncology, Center of Postgraduate Medical Education, 02-781 Warsaw, Poland; natalia.zeber-lubecka@cmkp.edu.pl; 4 Department of Genetics, Maria Sklodowska-Curie National Research Institute of Oncology, 02-781 Warsaw, Poland; 5 Second Department of Obstetrics and Gynecology, Center of Postgraduate Medical Education, 00-189 Warsaw, Poland; 6 Pediatric Ward, Department of Pediatrics, Center of Postgraduate Medical Education, Bielański Hospital, 01-809 Warsaw, Poland

**Keywords:** gut microbiota, gastrointestinal system, respiratory system, asthma, immunological diseases, atopic dermatitis, food allergy, newborn, premature newborn, antibiotic therapy

## Abstract

**Introduction:** The shaping of the human intestinal microbiota starts during the intrauterine period and continues through the subsequent stages of extrauterine life. The microbiota plays a significant role in the predisposition and development of immune diseases, as well as various inflammatory processes. Importantly, the proper colonization of the fetal digestive system is influenced by maternal microbiota, the method of pregnancy completion and the further formation of the microbiota. In the subsequent stages of a child’s life, breastfeeding, diet and the use of antibiotics influence the state of eubiosis, which determines proper growth and development from the neonatal period to adulthood. The literature data suggest that there is evidence to confirm that the intestinal microbiota of the infant plays an important role in regulating the immune response associated with the development of allergic diseases. However, the identification of specific bacterial species in relation to specific types of reactions in allergic diseases is the basic problem. **Background**: The main aim of the review was to demonstrate the influence of the microbiota of the mother, fetus and newborn on the functioning of the immune system in the context of allergies and asthma. **Methods**: We reviewed and thoroughly analyzed the content of over 1000 articles and abstracts between the beginning of June and the end of August 2024. Over 150 articles were selected for the detailed study. **Results**: The selection was based on the PubMed National Library of Medicine search engine, using selected keywords: “the impact of intestinal microbiota on the development of immune diseases and asthma”, “intestinal microbiota and allergic diseases”, “the impact of intrauterine microbiota on the development of asthma”, “intrauterine microbiota and immune diseases”, “intrauterine microbiota and atopic dermatitis”, “intrauterine microbiota and food allergies”, “maternal microbiota”, “fetal microbiota” and “neonatal microbiota”. The above relationships constituted the main criteria for including articles in the analysis. **Conclusions**: In the present review, we showed a relationship between the proper maternal microbiota and the normal functioning of the fetal and neonatal immune system. The state of eubiosis with an adequate amount and diversity of microbiota is essential in preventing the development of immune and allergic diseases. The way the microbiota is shaped, resulting from the health-promoting behavior of pregnant women, the rational conduct of the medical staff and the proper performance of the diagnostic and therapeutic process, is necessary to maintain the health of the mother and the child. Therefore, an appropriate lifestyle, rational antibiotic therapy as well as the way of completing the pregnancy are indispensable in the prevention of the above conditions. At the same time, considering the intestinal microbiota of the newborn in relation to the genera and phyla of bacteria that have a potentially protective effect, it is worth noting that the use of suitable probiotics and prebiotics seems to contribute to the protective effect.

## 1. Introduction

### 1.1. Allergic Diseases—Overview

Allergic diseases are a heterogeneous group of chronic inflammatory diseases. Analyzed individually, the group of allergic diseases includes food allergies, atopic dermatitis (AD) and hay fever [1,2]. The underlying cause of the conditions is a pathological, qualitatively altered response of tissues to the impact of various foreign substances, called allergens, consisting of an immune reaction associated with the formation of specific antibodies, which, after binding to an antigen, lead to the release of various substances, i.e., inflammatory mediators. Asthma is classified differently, as it may be divided into an allergic and non-allergic form based on the etiology [1,3,4,5].

Allergic diseases affect both the functioning and the economic status of families, society and the health care system [3,6,7,8,9,10,11]. The incidence of allergic and autoimmune diseases and asthma is constantly increasing [12,13,14,15,16,17]. It is estimated that at least 20–30% of the population of Western countries suffers from a form of allergic disease [6,18,19,20,21]. Almost 700 million people worldwide suffer from allergic respiratory diseases, e.g., bronchial asthma and allergic rhinitis (AR) [3,7]. It was found that allergic diseases are more common in children than in adults [3,7]. The number of children and adults suffering from allergic diseases is almost two times higher in urban areas compared to rural ones. This is due to the so-called “hygiene hypothesis” associated with a decrease in the activity of antigens in the environment, e.g., the widespread use of antibiotics, the distribution of vaccines, chemical additives in production, nutrition and a “sterile” lifestyle [21,22,23,24]. However, their etiopathogenesis is multifactorial. The impact of genetic [25,26], infectious [27,28,29], immune [30,31,32,33] and environmental [34,35,36] factors was repeatedly analyzed, and the above analyses provided the basis for subsequent scientific projects.

### 1.2. Intestinal Microbiota—Eubiosis/Dysbiosis

The state of eubiosis formed from the fetal period through further stages of extrauterine life is an important factor analyzed in the present review. Eubiosis is a regulator in the processes of predisposition to the conditions under consideration [37]. The disruption of the fetal intestinal microbiota may begin during intrauterine life due to the mother’s lifestyle, diet and medications, particularly antibiotic therapy [38,39]. The way of completing the pregnancy is another important factor influencing appropriate microbiota shape and predisposition to the development of allergic diseases [40,41]. This is a subject of current research.

**Figure 1:**
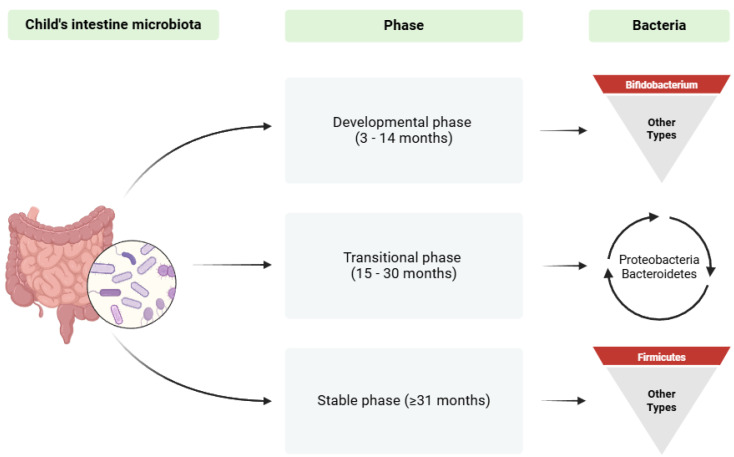
This figure shows the phases of development of an infant’s intestinal microbiota after birth. We distinguish the developmental phase between 3 and 14 months of age, in which bacteria of the *Bifidobacterium* species dominate; the transitional phase occurring between 15 and 30 months, in which significant quantitative changes occur in the Proteobacteria and Bacteroidetes phyla; and the stable phase (≥31 months), in which the Firmicutes phylum dominates. Notably, Stiemsma et al. identified the so-called “critical window” in which microbiome colonization had the greatest impact on the development of immunity. The period was found to include the first 100 days of life, when microbiota interventions might be the most effective [52]. However, taking account of the data on the fetal microbiota presented above, it should be assumed that this period should be extended to encompass intrauterine development. At the same time, when considering the development of fetal T lymphocytes, which progresses from the second trimester of pregnancy, it would be important to modulate the fetal microbiota from this period onwards.

Gut microbial dysbiosis causes metabolic and immune disorders, especially in infants and small children. It leads to metabolic, allergic and autoimmune disorders such as obesity [42], type 1 diabetes mellitus (T1D) [43], allergies [44], autism [45], non-specific inflammatory bowel disease (IBD) [46] and stunted growth [38]. The transformation of intestinal microbiota occurs rapidly within the first three years after birth. During that time, the composition of the microbiota becomes similar to that of an adult within the first year, while a stable community is achieved within approximately 3 years [47,48]. The shaping of the intestinal microbiota in terms of the stage and phyla may be examined based on three phases (Figure 1) [48,49,50,51].

Nevertheless, the basic problem is the precise identification of individual bacterial species. Therefore, this problem requires further intensive research so that appropriate modeling of the microbiota can lead to the greatest possible benefits for the fetus and newborn.

### 1.3. Correlations Between Allergic Diseases and Neonatal Intestinal Microbiota

Some authors showed a relationship between intestinal microbiota and immune activation in the intestines, systemic circulation, other organs and peripheral tissues [53].

This relationship was also demonstrated during pregnancy, when the maternal intestinal microbiota might affect the immune environment in the uterus. Intestinal dysbiosis occurring at that time was found to be associated with the occurrence of numerous conditions, such as gestational hypertension, gestational diabetes, premature delivery and recurrent miscarriages [54,55,56]. In addition, disorders of the maternal intestinal microbiota might lead to diseases in the later life of the offspring [57,58,59].

**Figure 2:**
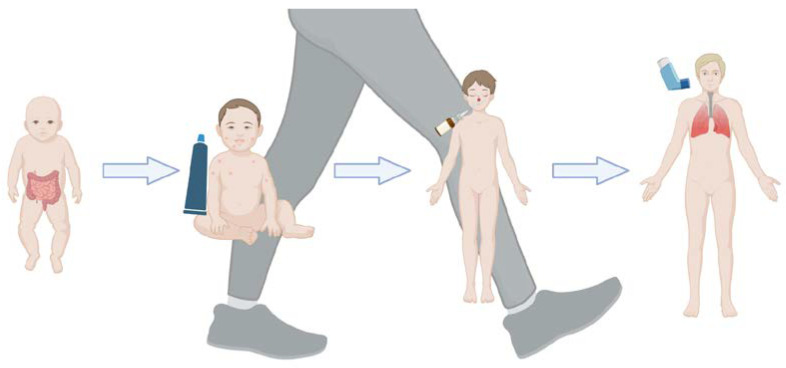
A model of the allergic march showing consecutive clinical problems ranging from AD to food allergy to AR to asthma. AD usually develops first on the basis of genetic predisposition and/or environmental factors. Due to the skin barrier dysfunction resulting from AD, the allergen penetrates the body, triggering epidermal sensitization and type 2 inflammation in some children. Then, memory Th2 (Th2) cells return to the skin and exacerbate AD. After that, they spread to the intestines, lungs and nose. Patients develop food allergies, allergic asthma and allergic rhinitis with increased sensitivity to food and/or environmental allergens, resulting in the induction of the atopic march.

When analyzing the relationship between microbiota and the occurrence of AD, attention was paid to a significant correlation (80%) between AD diagnosed in infants and young children and the development of AR and asthma later in life [60]. Confirming the above, Tanabe et al. cited the term used by Spergel et al., i.e., “the allergic march”, presenting the natural history of atopic manifestations, characterized by a typical sequence of the progression of allergic symptoms in infancy from AD and food allergy (FA) to asthma and AR [61,62] (Figure 2).

### 1.4. Asthma

Asthma is a chronic inflammatory disease of the respiratory system characterized by typical symptoms of periodic shortness of breath, coughing and wheezing [63]. In some industrialized countries, the incidence of asthma is approximately 35–40%, while in other regions, it is below 5% [3,64,65]. The prevalence of asthma is also increasing in numerous low- and middle-income countries [3,6,20,21,22,66].

The inflammatory reaction and sensitivity to allergens play the primary role in the pathophysiology of asthma [67]. The mechanism of immune response modulation in asthma has not been fully elucidated [40]. One hypothesis is based on the influence of metabolites of microbial origin produced in the form of short-chain fatty acids (SCFAs), with Lactobacilli being their main producer. SCFAs exert a protective and immunomodulatory effect by producing regulatory T cells (Tregs) from CD4 dendritic and precursor T cells and by activating the G-protein-coupled receptor (GPR) and inhibiting histone deacetylase [68]. Boro et al. noted that the placenta contained SCFAs and their receptors [69]. Research conducted on a murine model revealed that the transfer of antigen-specific IgG antibodies provided the offspring with protection against respiratory tract infections [70].

The role of the microbiota (both microorganisms and the microbiome) in the development of the inflammatory response is extremely important [71]. A link was found between prenatal factors, i.e., maternal smoking [72], maternal asthma history [73], intragestational antibiotic use [74], diet [75], maternal stress [76,77] and increased susceptibility to asthma. Dysbiosis with reduced microbial diversity and respiratory diseases were also shown to be associated [78]. It was noted that the dysbiosis of the gastrointestinal tract and respiratory system played a significant role in the pathophysiology of asthma by modifying the response of the immune system [79,80].

Some authors observed a relationship between bacteria obtained from the mother’s vagina and the presence of IgE in infants and allergic immunomodulation, which was an important factor in the development of asthma [44]. Very small amounts of IgE are usually found in the blood, and its concentration varies across age groups. However, low levels were also observed in newborns [81,82]. Several bacteria were identified that were associated with an increased susceptibility to asthma in the first year of life [67]. Conversely, material derived from meconium revealed the *L. jensenii* antigen, which, through the immunosuppressive effect, could modulate the function of the fetal immune system in the uterus in a way that might promote increased immune tolerance to allergy in infancy [66]. Continuing research on mice in which microorganisms intended to protect against asthma were supplemented, a reduction in the inflammation of the respiratory tract was demonstrated, which may confirm the hypothesis of the role of the infant’s microbiota in the development of asthma [68].

Nevertheless, identifying a specific taxonomic group associated with the development and severity of asthma is still a considerable challenge. At the same time, some authors attempted to demonstrate a relationship and influence of genetic factors on shaping the human intestinal microbiota [83]. Similarly, several studies revealed a relationship between blood groups (A, B antigens) and characteristic phyla of bacteria [84,85,86].

### 1.5. Atopic Dermatitis

According to the literature, 60% of AD cases occurred as early as in the first year of life, presenting as infantile eczema [87]. A number of factors were shown to affect the development of AD. Nevertheless, the latest research, which is gaining attention, raised the problem of intestinal dysbiosis and its regulatory mechanism in the processes of immune system functioning. The production of regulatory T cells was highlighted due to their protective activity against inflammatory processes that trigger disorders common in allergic and autoimmune diseases [88,89,90].

Previous research on the impact of specific bacterial taxa in combination with allergic diseases gave rise to the hypothesis that it was the reduced diversity of intestinal microbes, and not the presence or abundance of individual taxa, that increased the risk of allergic symptoms in childhood [1]. The above hypothesis was associated with an increased risk of AD [91,92,93]. However, different cohort studies demonstrated that less marked bacterial diversity was associated with AR and sensitization, but not with AD [1]. A similar view was presented by McCauley et al. [94]. They found that a rich vaginal environment was associated with a reduced incidence of allergies in children. Conversely, the incidence increased with the domination of selective *L. fornicalis* or *G. vaginalis* species by stimulating the production of lipopolysaccharide, causing vaginal dysbiosis and inflammation [95,96]. Similarly, the dominance of the vaginal flora by *Lactobacillus* was associated with the infant’s IgE level in the first year of life, which was related to susceptibility to asthma [94]. Similarly, in relation to the extrauterine life, individuals’ own behaviors, modeling the gastrointestinal environment and resulting in dysbiosis, were found to expose them to the occurrence and multiplication of the incidence of the above-mentioned diseases [38,39]. Therefore, exposure to parasites and microorganisms and related treatment constitute important factors increasing the excessive reactivity of the immune system and the development of allergic diseases [24].

Nylund et al. noted greater bacterial diversity in the fecal microflora of infants with allergic eczema [97]. The differences might result from methodological variations both in the populations covered by the study and the implementation of techniques used to identify bacteria, as well as the time of collecting material from the subjects. Therefore, it seems reasonable to collect material at an early stage of development at the time of birth, i.e., during the maturation of the immune system and the development of immune tolerance to food antigens and microorganisms [98,99]. Considering the above, it would be valuable to conduct an analysis of the impact of maternal microbiota on the microbiota, development and functioning of the fetus and the newborn. Conducting such a study would be justified, as the analyzed materials clearly indicated the influence of factors such as nutrition [100,101], administration of probiotics or antibiotics [102,103] and the mode of delivery [104,105,106] on the composition of the developing microbiota, early stages of colonization and commensal intestinal microflora, i.e., factors shaping the immune system and potentially affecting the development of various diseases [107].

### 1.6. Food Allergy

It is currently estimated that about 10% of the world’s population exhibits signs of food allergy. The incidence is a significant and constantly growing problem [108].

It has been repeatedly mentioned that the state of intestinal eubiosis might also constitute a protective factor against food allergies by promoting the development of the immune system [109,110,111,112]. Notarbartolo et al. identified the types of bacteria showing protective properties by stimulating the immune response of Th1 helper lymphocytes (Th) and inhibiting the immune response of the Th2 type, as well as bacteria promoting allergic reactions [113].

Shuo et al. analyzed the differentiation of bacterial phyla depending on age after birth, determining the method of changes in bacterial abundance over time in children with food allergies [114]. At the same time, studies by Wang et al., Notarbartolo et al. and Gao et al. presented a similar conclusion in relation to *Holdemania* spp., indicating the correlation of its abundance in the mother’s stool with the occurrence of food allergies in the child [41,113,114]. Moreover, a correlation between polyunsaturated fatty acids and the amount of *Holdemania* spp. was also analyzed. Therefore, this type of bacteria was found to be associated with a reduced risk of food allergy in the offspring and, thus, could be used as a predictive marker [113,114].

The main aim of the review was to demonstrate the influence of the microbiota of the mother, fetus and newborn on the functioning of the immune system in the context of allergies and asthma.

## 2. Materials and Methods

We reviewed and thoroughly analyzed the content of over 1000 articles and abstracts between the beginning of June and the end of August 2024. Over 150 articles were selected for the detailed study. The selection was based on the PubMed National Library of Medicine search engine, using selected keywords: “the impact of intestinal microbiota on the development of immune diseases and asthma”, “intestinal microbiota and allergic diseases”, “the impact of intrauterine microbiota on the development of asthma”, “intrauterine microbiota and immune diseases”, “intrauterine microbiota and AD”, “intrauterine microbiota and food allergies”, “maternal microbiota”, “fetal microbiota” and “neonatal microbiota”. We focused on searching for information on the correlation between fetal and neonatal microbiota and the functioning of the immune system in terms of allergies and asthma. Due to the considerable relationship between the mother, the fetus and the newborn, we also described additional factors modulating immune processes, taking account of the maternal microbiota and the method of completing the pregnancy. The above relationships constituted the main criteria for including an article in the analysis. The exclusion criteria were as follows: no specific data concerning the child’s age, no exact time of the onset of disease symptoms and no specified time from birth to the collection of the material (meconium/stool) for examination from a newborn. The analysis also did not include children after the neonatal period. In the article, we do cite information on the population of children beyond the neonatal period, but the analysis is not based on this group. This information is only included due to the need to pay attention to the broader context of changes occurring in the child’s body.

## 3. Synthesis of Studies Included in the Systematic Review

### 3.1. Microbiota and the Immune System of the Fetus and Newborn

**Figure 3:**
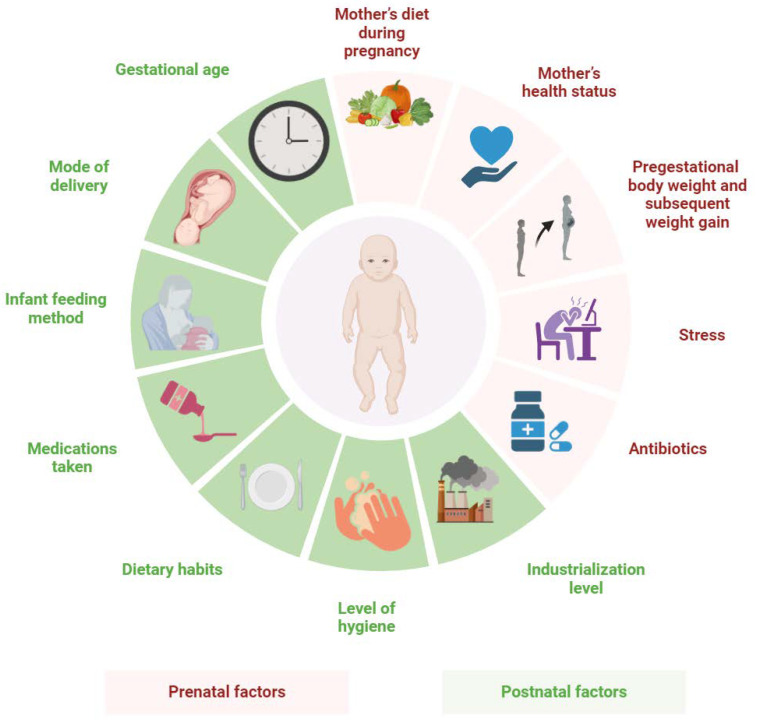
The most common prenatal and postnatal factors influencing the intestinal microbiota and the immune system of the fetus and newborn.

Adamczak et al. described the modulation of the fetal immune system by bacterial strains obtained from the mother through the placenta [115]. Similarly, Lloyd-Price et al. determined that the intestinal microbiota was necessary to shape the immune system as a result of the presence of bacteria that promoted immune tolerance, thereby reducing the incidence of autoimmune diseases and food allergies [116]. The dynamics of microbiota development, which changes throughout life in response to various internal and external factors, are equally important [117]. An important role is played by some components, i.e., the mother’s diet during pregnancy [118,119,120], the mother’s health status [121], pregestational body weight and subsequent weight gain [122], stress [123] and the use of antibiotics [124]. Postnatal factors include gestational age [125], mode of delivery [126], method of feeding infants [126], medications administered to the child at later stages of life [127,128,129], eating habits [129,130] as well as the level of hygiene and industrialization of the environment [131,132] (Figure 3). Similarly, Miyoshi et al. identified maternal variables such as antibiotic use, diet, obesity and diabetes in association with changes in the formation and evolution of the offspring’s intestinal microbiota and their impact on fetal and neonatal immunity [133].

The confirmation of the above hypotheses may also be found in studies by Gomez de Agüero et al., Mueller et al. and Suárez-Martínez et al., who discussed the interaction between the maternal microbiota and the fetal immune system during pregnancy and the presence of intestinal microorganisms in the placenta, amniotic fluid and fetal membranes [134,135,136]. Research by Indrio et al. showed that the nutritional status of the mother played a key role in developmental programming and changing the risk of non-communicable diseases in the offspring through epigenetic modifications [137].

A study by Qian et al. showed that the composition of the microbiota in early life changed the balance of Th1/Th2 helper lymphocytes, thereby influencing the risk of allergy [138]. The above hypothesis was confirmed by Stencel-Gabriel et al. and Nyangahu et al. They reported on the association of the colonization of the mother’s vagina by *Lactobacillus* with reduced levels of interleukin IL-12 (a factor enhancing the response of NK (natural killer) cells and macrophages) in the umbilical cord blood after delivery. Lactobacilli also exerted a significant influence on the modulation of T cell development in newborns. They noted that CD4+ T helper cells (CD4+) and CD8+ T lymphocytes (CD8+) were present in the fetus at the end of the first trimester. They produced IL-2 and tumor necrosis factor (TNF)-α. They also supported intestinal development. This suggests that the presence of *Lactobacillus* bacteria in the mother’s vagina might have an impact on the development of the fetal immune system [139,140].

According to Donal et al., the immune system was immature after birth and mainly depended on congenital factors. Neonatal dendritic cells were found to exhibit adult levels of the immunoregulatory IL-10 cytokine after stimulation with lipopolysaccharides (LPS) obtained from the maternal microbiome. However, they were less effective in promoting Th1 differentiation due to delayed IL-12 production. The immune system of newborns, by promoting immunoregulatory responses and Th2, shaped a protective mechanism to prevent excessive inflammatory responses to new antigens occurring in the environment and commensal microorganisms from their own microflora [141].

However, with regard to the microbiome, which is directly involved in the processes presented above, Bokulich et al. described the composition of the intestinal microbiota in the period between the first and seventh day of life of a newborn, dominated mainly by Proteobacteria, Firmicutes, Bacteroidetes and Actinobacteria [142]. Ygberg et al. highlighted an important aspect of the correlation between the above microbiota modification and the simultaneous increase in the number of neutrophils with the maturation of monocytes and macrophages in the neonatal intestines, which are fully developed around the seventh day after birth [143].

In the present analysis, we noted an inseparable relationship between the maternal microbiota and its impact on the child’s immune system. The above correlation indicated the need to maintain the state of eubiosis in order to properly modulate the activity of the immune system and prevent inflammatory processes in the offspring. Therefore, proper health-promoting behavior, i.e., both physical activity and the mother’s diet, is an important factor influencing the proper functioning of the child’s immune system.

### 3.2. Microbiota and the Method of Completing the Pregnancy

The colonization of the newborn’s gastrointestinal tract is closely related to the mode of delivery and gestational age at birth [144]. According to Kalbermatter et al., a child born vaginally has direct contact with microorganisms colonizing the intestinal lumen and vagina of the mother, i.e., bacteria of the genus *Lactobacillus*, *Bacteroides* and *Bifidobacterium* [145]. Therefore, the microflora is similar to that of the mother [146,147]. The above hypothesis was confirmed by Méndez et al., who showed that newborns born vaginally showed a greater abundance of *Bifidobacterium*, Bacteroides, *Lactobacillus* and bacteria belonging to the *Lachnospiraceae* family and bacteria originating from the vagina (i.e., *Lactobacillus* spp. and *Prevotella* spp.) [148]. A study by Mesa et al. demonstrated the presence of Sneathia spp. in addition to *Lactobacillus* and *Prevotella* [149].

Yao et al. noted the presence of *Clostridiaceae*, *Enterococcaceae* and *Streptococcaceae* families in neonates in the first days after vaginal birth, while Bacteroides and *Bifidobacterium* appeared in the intestines of 40% of infants from the third day onwards after birth. *Bifidobacterium* became dominant, and its relative abundance was the highest on days 4–7 [147]. Coelho et al. demonstrated that, within the first 24 h after birth, the baby’s digestive tract was colonized by *Staphylococcus*, *Lactobacillus* and *Enterococcus*. These bacteria were responsible for activating immune cells and preparing the intestinal environment for the growth of anaerobic bacteria (Table 1) [150].

Based on the above summary presenting the dominant intestinal microbiota of newborns, it can be seen that in each case the genus *Lactobacillus* was detected (Table 1), in three cases *Bacteroides* and *Bifidobacterium* and in single studies *Enterococcaceae*, *Streptococcaceae*, *Clostridiaceae*, *Lachnospiraceae*, *Prevotella*, *Staphylococcus* and *Sneathia* spp.

As regards the colonization of the upper respiratory tract, Bosch et al. reported that it began with *Staphylococcus* and *Streptococcus*, followed by the proliferation of *Moraxella*, *Corynebacterium*, *Dolosigranulum* and/or *Haemophilus* species [151], which were associated with a reduced risk of respiratory symptoms [152,153]. Conversely, they noted a lower number of *Corynebacterium* and *Dolosigranulum* in the upper respiratory tract of newborns born by cesarean section in the first months of their lives [151].

To compare, newborns born by cesarean section had about 30% fewer bacterial species than their mothers [145]. In the intestinal microbiota of newborns, Rutavisire et al. showed the presence of microorganisms from the mother’s skin and antibiotic-resistant bacteria from the hospital environment, including *Staphylococcus*, *Streptococcus* and *Clostridium* [126]. Conversely, Gabinelli et al. and Méndez et al. determined that *Enterococcaceae*, *Enterobacteriaceae* and maternal skin microorganisms (i.e., *Staphylococcus* spp. and *Propionibacterium* spp.) were the most abundant in children born by cesarean section [148,154]. Similar conclusions were proposed in a study by Rachida et al. [155]. As regards neonates born by cesarean section, Eun et al. determined a lower level of Bacteroides bacteria, less diversity within the Bacteroidetes phylum and a higher level of diversity within the Firmicutes phylum (Table 2) [156].

Based on the above summary presenting the dominant intestinal microbiota of newborns, it can be seen that in four cases the genus *Staphylococcus* was detected (Table 2); in three cases *Enterococcaceae*, *Enterobacteriaceae* and *Propionibacterium*; and in single cases *Streptococcus*, *Clostridium*, Bacteroidetes and Firmicutes.

When analyzing the intestinal microbiota of a newborn, depending on the method of pregnancy completion, it may be seen that the Firmicutes phylum predominated with the advantage in the *Lactobacillus* family after vaginal delivery (Table 1 and Table 2). The presence of Bacteroidetes and *Acinobacteria* was also noticeable, with the families *Bacteroidaceae* and *Prevotellaceae* for Bacteroidetes and *Bifidobacteriaceae* for Actinobacteria. A small amount of *Fusobacteria* was also noted. After cesarean section delivery, the presence of the families *Lachnospiraceae* and *Lactobacillaceae* of the Firmicutes phylum, the *Prevotellaceae* family of the Bacteroidetes phylum and the *Leptotrichiaceae* family of the *Fusobacteriota* phylum was not confirmed. Conversely, a significant amount of the *Enterobacteriaceae* family of the Proteobacteria phylum and the *Propionibacteriaceae* family of the Actinobacteria phylum was identified. Moreover, they were not found after vaginal births.

### 3.3. Correlation of Various Factors Predisposing to Asthma

Genetic factors, the immune system, the course of pregnancy, antibiotic therapy in the mother and child, and the mode of delivery affect the composition and function of the microbiota in both the intestines and lungs [157].

Asthma is a polygenic, multifactorial disease. Its development is influenced by numerous factors, both genetic and environmental [158]. The risk of developing the disease depends on the degree of genetic relationship between the child and the relative with the disease. The risk is usually higher if the relative is severely affected, or if the relative was affected at an early age. Unlike single-gene disorders, the asthma phenotype is characterized by nonlinear expression and high variability. Therefore, it is difficult to predict asthma status for a given genotype or a combination of genotypes [159]. Hammad et al. identified two main forms of asthma: allergic and non-allergic asthma. However, this was found to be oversimplified. Allergic asthma tends to start in childhood and is associated with Th2 cell responses that also occur in other allergic conditions such as AD or AR. It is caused by early contact with environmental allergens. The onset of the disease coincides with an important period of development of the immune and structural system of the lungs in early childhood. Lifelong homeostasis and susceptibility to immune-mediated diseases, such as asthma, are formed in the neonatal period [160]. According to Pakkasel et al., in contrast to allergic asthma, non-allergic asthma usually occurs later in life and is more common in women and obese patients [161]. At the same time, research by Loewen et al., Stensballe et al., Metsälä et al. and Mulder et al. showed an increased risk of asthma in children exposed to antibiotics prenatally [162,163,164,165]. Conversely, Roduit et al. observed a higher risk of asthma in children born by cesarean section compared to vaginal births [166]. Nevertheless, regardless of the factors predisposing to asthma, most authors highlighted their impact on intestinal dysbiosis as a superior factor.

#### 3.3.1. Genetics and Asthma

A study by Qian et al. showed that the composition of the microbiota in early life changed the balance of Th1/Th2 helper lymphocytes, thereby influencing the risk of developing an allergy. However, Th2 asthma is characterized by an increased activation of Th2 in response to allergens, leading to the accumulation of eosinophils in the lungs, causing inflammation of the respiratory tract by increasing the production of immunoglobulin E (IgE) [138]. Zhang et al. and Han et al. noted that oral intake of *L. rhamnosus* and *L. murinus* promoted the migration of Tregs to the lungs and blocked Th2 response, which reduced airway inflammation [167,168]. Wang et al., based on an OVA murine model, also confirmed that *L. fermentum* could reduce the expression of Toll-like receptor 2 and Toll-like receptor 4, while reducing the infiltration of inflammatory cells and swelling of the alveoli [169]. In contrast, with respect to correlations between intestinal microbiota, host genetics and asthma, Li et al. reported that, based on a Mendelian randomization analysis of two samples, they predicted a positive correlation between *Barnesiella* intestinal species and *Ruminococcaceae* UCG014 genera and the risk of asthma. They also found that *Akkermansia* reduced the risk of asthma in adults [170]. Perez-Garcia et al. conducted an analysis which linked the polymorphisms of the microbiome quantitative trait loci (mbQTL) with the presence of *Streptococcus*, *Tannerella* and *Campylobacter* in the upper respiratory tract. They identified diseases concomitant with asthma through polymorphisms in the genes APOBEC3B-APOBEC3c, TRIM24 and TPST2 [171]. Boulund et al. identified correlations between host genes and microbial taxa and their association with secretory metabolism, signaling transport and immunity [172]. Similarly, Lopera-Mayá et al. conducted a genome-wide association study to determine the influence of host genetics on the intestinal microbiome. They found two sites near the lactase genes and the ABO group for the genera *Bifidobacterium* and *Collinsella*, respectively [84]. Rühlemann et al. reported a relationship between the *Prevotella* phylum in asthma and ABO blood groups [85]. Ahluwalia et al. observed that changes in Fucosyltransferase 2 (FUT2) and ABO genes might contribute to an increased risk of asthma in early childhood by expressing AB antigens in the respiratory tract epithelium and *Streptococcus pneumoniae* infection [86]. Conversely, Kurilshikov et al. conducted research on the impact of human genetic variability on microbial taxa. They identified 31 loci that affected the intestinal microbiome, including the LCT gene loci, which are important for the genus *Bifidobacterium* [173].

A correlation was observable between characteristic gene loci with specific microbiota species and subsequent inflammatory reactions, including asthma. Therefore, the appropriate composition of the microbiota is a basic protective factor against the occurrence of asthma, due to the lack of the combination of characteristic bacterial species in specific airway loci. In this aspect, it is the only possibility to prevent the development of asthma.

#### 3.3.2. Prematurity and Asthma

According to the World Health Organization (WHO), preterm birth is defined as any delivery before 37 weeks of gestation or before 259 days from the first day of the last menstrual period (LMP) of a woman. It is subdivided based on gestational age (GA):

- Extremely preterm (<28 weeks);
- Very preterm (28–<32 weeks);
- Moderate or late preterm (32–<37 completed weeks of gestation) [174].

According to Zimmermann et al., preterm birth may lead to delayed and limited colonization of the intestines by beneficial bacteria such as *Bacteroides*, *Bifidobacterium* and *Lactobacillus*. This may result in an increased possibility of the colonization of the intestine by *Clostridiaceae* and the following risk of developing asthma and allergic diseases [175]. A meta-analysis by Zhang et al. showed that premature infants were at a 36% higher risk of asthma compared to full-term infants [176]. Cump et al. described that the risk of developing asthma increased with a decrease in the gestational age at birth, with a probability 1.5 to 2.5 times higher compared to a full-term newborn [177]. Arroyas et al. associated the above with poorer function, as well as structural changes in the lungs of a premature infant [178]. Raita et al. identified an endotype characterized by several typical features including a high incidence of asthma in parents, IgE allergy and concomitant rhinovirus (HRV) infection. The co-dominance of *Streptococcus pneumoniae* and *Moraxella catarrhalis* in the nasopharynx, along with an increased response to IFN-α and -γ, also constituted dominant features. Preterm infancy was reported in approximately 22.2% of the above population [179]. The above relationship was confirmed by Bizzintino et al., who claimed that HRV-C infection was associated with more severe asthma exacerbations compared to HRV-A and HRV-B [180]. Pinto et al. noted that the above infections reduced the Th1 and IFN-γ responses, leading to the intensification of the Th2 response conducive to inflammation and bronchoconstriction [181]. Similarly, Chesné et al. and Anderson et al. observed elevated levels of IL-17 from Th17 cells in premature infants, which further exacerbated inflammation, resulting in both eosinophilic (IL-4 and IL-13) and neutrophilic (IL-17) asthma, leading to a mixed type of asthma [182,183]. At the same time, the mode of delivery of premature babies (infants born before 37 weeks of gestation), which very often involves a cesarean section, was associated with the development of various health issues, including asthma.

As regards prematurity, the immature immune system and the associated dysbiosis aggravated by the frequent use of antibiotic therapy and cesarean section as a method of completing the pregnancy might underlie immune system disorders contributing to the development of asthma. Therefore, it is necessary to conduct rational antibiotic therapy in premature infants, reserving it only for conditions where it is required. Also, the premature completion of pregnancy by means of cesarean section should be reserved only for life-threatening conditions of the mother or child.

#### 3.3.3. Relationship Between the Mode of Delivery and Asthma

According to Salas Garcia et al., Ferllini Montealegre et al. and Kumbhare et al., changes in the microbiota during cesarean section, especially in the elective mode, were associated with changes in the immune system, increasing the risk of developing asthma, allergies, type I diabetes and celiac disease [184,185,186]. This was confirmed by Thavagnanam et al. and Bager et al. [187,188]. Tollanes et el. observed stronger correlations between cesarean section and the risk of the development of asthma in the case of emergency cesarean sections compared to elective ones [189].

Słabuszewska-Jóźwiak et al. determined the odds ratio of asthma development in children to be 1.23, with a higher incidence of asthma being observed in children born by cesarean section [190]. Similarly, Moore et al. observed the relative risk to be 1.11 in children with partially controlled asthma and 1.8 in those with uncontrolled asthma [191]. Penders et al. showed that newborns born by cesarean section were more often colonized by *Clostridium difficile* [192]. Similarly, Van Nimwegen et al. linked the mode of delivery with intestinal microbiota (*C. difficile*) and asthma [104].

At the same time, Li et al., Kozyrskyj et al., Thavagnaman et al., Jakobsson et al., Hwang et al. and Bach et al. demonstrated an increased risk of asthma and allergic reactions associated with cesarean section through the reduced abundance and diversity of bacteria with reduced colonization by protective bacteria and, consequently, an increased number of potential pathogens, with a low level of Th1 response [187,193,194,195,196,197]. In contrast, Khafipour et al., Dominguez-Bello et al. and Bisgaard et al. identified the increased diversity of intestinal microbiota associated with vaginal births as a preventive factor for the development of asthma and allergies [198,199,200].

To sum up, we noted that the reduction in the abundance and diversity of bacterial species following a cesarean section promoted the excessive accumulation of pathogens, negatively influencing the development of inflammatory reactions. Vaginal delivery was found to have a different impact, fostering the diversity of bacteria. Therefore, the right approach to indications concerning the method of completing the pregnancy is a key element in preventing the development of asthma.

#### 3.3.4. Antibiotic Therapy and Asthma

**Figure 4:**
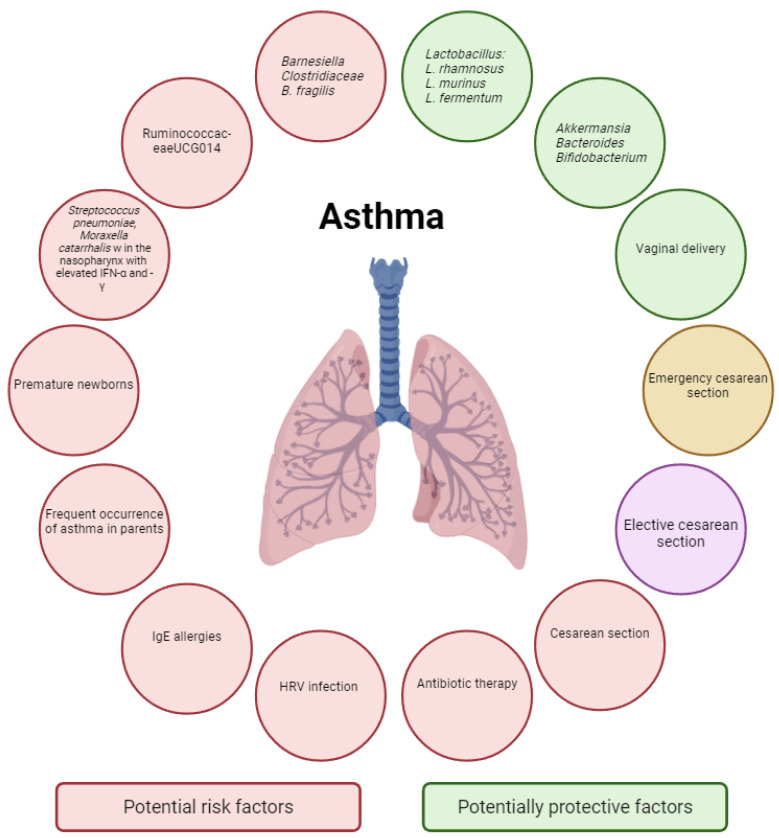
The diagram shows factors which potentially influence the development of asthma. Risk factors for asthma are marked in red, while potentially protective factors are marked in green. As regards cesarean sections, we may distinguish elective and emergency cesarean sections. Due to the greater number of reports on the negative impact and promotion of the risk of developing asthma in the case of elective cesarean sections, it was marked in purple. Conversely, cesarean section due to emergency indications was marked in yellow to indicate that it was a potentially protective factor compared to the planned procedure.

The relationship between *C. difficile* and the risk of developing asthma was also indirectly demonstrated by Mai et al. and Jedrychowski et al. They based their research on correlating antibiotic therapy in early life with the risk of wheezing or asthma as a factor disturbing the intestinal microbiota of newborns (Figure 4) [201,202]. Similarly, Russell et al. conducted a study in an animal model and determined greater susceptibility of mice to asthma if their intestinal microbiota was disturbed by the use of vancomycin [203]. Galeana-Cadena et al. and Stiemsma et al. also stated that the administration of antibiotics close to the date of delivery had a significant impact on the diversity of the microflora of both the newborn and the mother. An increase was observed in the abundance of the genus Proteobacteria compared to the dominant phylum Firmicutes with families such as Streptococcaceae and Lactobacilaceae in newborns whose mothers did not receive antibiotic therapy [83,204]. Vael et al. identified the subgroup *Bacteroides fragilis* and the subclass XIVa *Clostridium coccoides* in the intestinal microbiota of infants to be early indicators of asthma risk later in life [205].

To conclude, the intestinal microbiota is disturbed by the excessive use of antibiotic therapy. Therefore, the only method of preventing bacterial dysbiosis is rational antibiotic therapy.

### 3.4. Microbiota and Atopic Dermatitis

Recent reports have indicated that the intestinal microbiota of the pregnant mother plays an important role in the development of fetal immunity and allergic diseases in the offspring [41,206,207,208]. Tanabe et al. observed that the reduced diversity of Proteobacteria and the relative abundance of Actinobacteria in pregnant women were significantly related to the development of allergies in infancy [61]. This was confirmed in studies by Zimmermann et al. and Wang et al. concerning the diversity of intestinal microbiota in children with AD, which demonstrated their low value [93,175]. At the same time, Zimmermann et al. reported that Clostridium (the Firmicutes phylum) and Enterobacteriaceae (the Proteobacteria phylum) were more abundant compared to *Bifidobacterium* (the Actinobacteria phylum) and *Lactobacillus* (the Firmicutes phylum) [175]. Nevertheless, the time discrepancy regarding the collection of fecal material and differences in microbiological diagnostic methods were limitations of the above studies [175,209].

Tanabe et al. also demonstrated a structural separation of the bacterial flora found in the feces of women at 12 and 32 weeks of pregnancy, indicating the dominance of four phyla. The dominant phyla included *Firmicutes*, Bacteroidetes, Actinobacteria and Proteobacteria [61]. Similarly, Fan et al. identified two dominant types, i.e., Firmicutes and Bacteroidetes, with a greater abundance of Bacteroides in the mothers of infants with AD and a reduced abundance of *Prevotella* [209].

**Figure 5:**
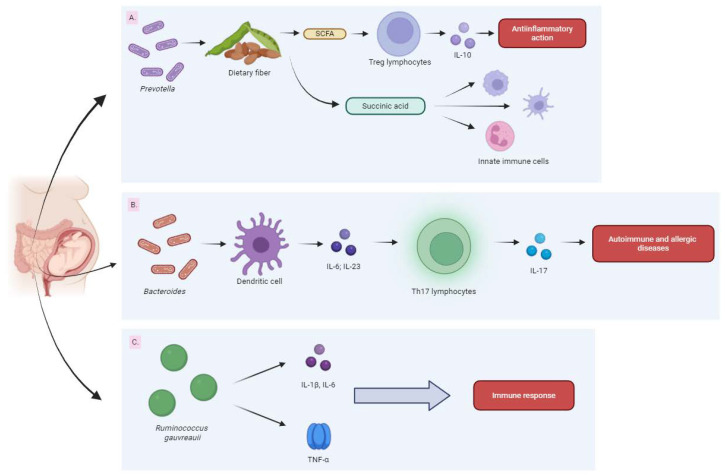
The diagrams show (**A**) a potential effect of *Prevotella* in the gastrointestinal tract of pregnant women and newborns on the prevention of childhood allergic diseases, (**B**) a potential activity of Bacteroides in the gastrointestinal tract of pregnant women and newborns promoting the development of autoimmune and allergic diseases in children, and (**C**) a potential activity of *Ruminococcus gauvreauii* in the gastrointestinal tract of pregnant women and newborns promoting the development of autoimmune and allergic diseases in children.

Therefore, it was concluded that increasing the abundance of *Prevotella* during pregnancy might serve as a protective factor against the development of childhood allergic diseases. Additionally, Vuillermina et al. showed that an increased abundance of *Prevotella* in pregnant women was associated with a reduced risk of developing allergies in their offspring [208]. Considering the mode of action of *Prevotella*, which consists of the fermentation of dietary fiber to produce metabolites, including SCFA and succinic acid, it should be noted that SCFAs exhibited an anti-inflammatory activity through the production of regulatory T cells producing interleukin-10 (IL-10) [89,210,211]. Succinic acid may stimulate the development, migration and function of innate immune cells of the fetus (Figure 5, item A) [212].

Conversely, Kamada et al. described the effect of Bacteroides promoting the secretion of IL-6 and IL-23 by dendritic cells, thereby influencing the differentiation of Th17 lymphocytes and the secretion of IL-17 [213]. Consequently, Th17 cells increased the risk of developing chronic autoimmune and allergic diseases in children by triggering inflammatory pathways (Figure 5, item B) [214].

A study by Xu et al. showed that the *Ruminococcus gauvreauii* species found in the intestinal microbiota exerted an impact on the mechanisms of the systemic immune response by proinflammatory cytokines including tumor necrosis factor alpha (TNF-α), IL-1β and IL-6 (Figure 5, item C) [215].

Fan et al. collected fecal materials perinatally for a study concerning the relationship between the intestinal microbiota of the mother and the offspring with the subsequent development of AD in infants and young children. They showed a relationship between AD and the presence of *Candidatus stoquefichus* and Pseudomonas occurring in significant amounts during pregnancy. They showed differences in pathogens prevalent in different age groups: *Eubacterium xylanophilum* at birth, *Ruminococcus gauvreauii* at 1 year of age and *UCG-002* at 2 years of age, with a concomitant decreased abundance of *Gemella* and *Veillonella* at 2 years [209].

The above review showed that Firmicutes was the dominant phylum of bacteria in children with AD. Differences may only be identified in species depending on age groups. Species of the phylum Bacteroidetes, i.e., *Prevotella* and *Bacteroides*, exert a different effect. *Prevotella* is a factor that prevents the development of inflammatory reactions and autoimmune and allergic diseases that follow, including AD. Conversely, *Bacteroides* promotes the above processes. It should be concluded that, in the case of the Bacteroidetes phylum, it is not the type but the species diversity that plays the main role in the prevention or promotion of AD. Furthermore, it may be noted that proper shaping of the intestinal microbiota by increasing the abundance of the *Prevotella* species may facilitate the prevention of AD.

### 3.5. Microbiota and Food Allergy

Renz et al. and Di Costanzo et al. determined that intestinal dysbiosis, i.e., an imbalance in the intestinal microbiome, preceded the onset of food allergy [216,217]. For a clearer discussion, the issue will be presented in separate subsections.

#### 3.5.1. Food Allergy and Intestinal Dysbiosis in the Course of Antibiotic Therapy

According to Molloy et al., the allergic response is increased by an early change in the composition of the intestinal microbiome, associated with the premature disruption of the intestinal epithelial barrier [218]. Conversely, Azad et al. and Augustine et al. demonstrated the dependence of food allergy on the decrease in the diversity of the intestinal microbiome [219,220] including an increased abundance of Enterobacteriaceae and a reduced abundance of *Bacteroidaceae* and *Ruminococcaceae* [219]. According to Zhang et al., antibiotic therapy was an important risk factor for intestinal dysbiosis in the mechanism of changing the diversity of the intestinal microbiome [221]. Samarra et al. claimed that the intestinal environment of newborns offered lower resistance to colonization, which potentially facilitated the emergence of antibiotic-resistant populations. Factors correlated with an increased incidence of antibiotic resistance genes included the use of antibiotics during childbirth and in the neonatal period [222]. Wang et al., Vuillermin et al. and Molloy et al. determined the relationship between the reduced abundance of *Prevotella* and *Bifidobacterium* as a result of, for example, exposure of a pregnant woman to antibiotic therapy, and the risk of allergic diseases [114,208,218]. Zhang et al. provided an indirect confirmation of the above in a study conducted on a murine model. Research showed a correlation between local and systemic inflammation caused by the dysbiosis of the intestinal microbiota, leading to damage to the intestinal barrier [221]. Similarly, Cheng et al. demonstrated a significant role of intestinal commensal bacteria in the modulation of immune tolerance by reducing the population of circulating basophils, promoting the integrity of the epithelial barrier (microbiological signals are able to modulate the production of mucus, mucin and occludin) and inducing the differentiation of Treg cells [223].

#### 3.5.2. Food Allergy, Maternal Diet and Its Impact on Intestinal Dysbiosis

Maternal diet is an additional factor affecting the modulation of the intestinal microflora. Vuillermin et al. discussed the impact of maternal diet and the presence of *Prevotella* during pregnancy on the likelihood of food allergy development in the offspring. They observed a significant relationship between the amount of *Prevotella* in the mother’s stool and a reduced risk of food allergies in 12-month-old infants [208]. According to Wu et al. and Fava et al., a high-fat diet increased the total anaerobic microflora and the abundance of *Bacteroides* spp. and reduced the abundance of *Bifidobacteria* spp. in the feces [224,225]. Ling et al. and Rey-Mariňo et al. studied the characteristics of the intestinal microbiota of children diagnosed with food allergies. It was characterized by reduced *Bacteroides* spp., *Bifidobacteri* spp. and *Clostridi* spp., with a consistent abundance of *Anaerobacter* spp. [226,227]. At the same time, Azad et al. showed a slight species diversity of the intestinal microbiota in the above population of children with the relative abundance of *Bacteroides* spp. [219]. A study by Gao et al. made an important finding about the protective effect of the diversity and richness of the intestinal microbiota, i.e., *Holdemania* spp., *Roseburia* spp., *Lachnospira* spp. and *Coprococcus* spp., in relation to the risk of developing allergic diseases [41]. Wang et al., Notarbartolo et al. and Gao et al. presented a similar conclusion in relation to *Holdemania* spp., indicating the correlation of its abundance in the mother’s stool with the occurrence of food allergy in the child [41,113,114]. The authors also associated the presence of *Holdemania* spp. with polyunsaturated fatty acids. This type of bacteria was found to be associated with a reduced risk of food allergy in the offspring. Therefore, it might be used as a predictive marker [113,114].

Shuo et al. analyzed the stool microbiota of mothers and newborns immediately after delivery. They showed the main phyla were Firmicutes, Actinobacteria, Proteobacteria and Bacteroidetes [114]. In the following months of the child’s life, they noted a stable number of Proteobacteria and Bacteroidetes, with variability in Actinobacteria and Firmicutes, which showed an increasing trend in the infant’s early life. The breakthrough period occurred at 6 months of age, when the abundance of Actinobacteria increased in children with food allergies, and a downward trend was noted in the case of Firmicutes. Considering the type of bacteria in the group of children with food allergies, *Bacteroides* and *Prevotella* constituted the highest percentage. However, their abundance was still relatively small compared to the control group. A much lower percentage of *Escherichia*/*Shigella*, *Clostridium* XIVa, *Faecalibacterium*, *Parabacteroides* and *Ruminococcus* was noted, but their abundance was greater in the group with food allergies compared to the control group [114].

Based on the presented analysis, we noted that dysbiosis of the intestinal microbiota was the primary mechanism conducive to the occurrence of food allergies, with environmental issues, medical problems and dietary habits being the essential contributing factors. Therefore, it is important to prevent intestinal dysbiosis and maintain the diversity of the intestinal microbiome in pregnant women by appropriate and rational nutrition, as well as the use of antibiotic therapy without its escalation. The above data also suggest that the use of appropriate dietary supplementation during pregnancy may support the maintenance of eubiosis, i.e., it is a protective factor against allergic diseases.

When developing data from the literature and creating the above article, it can be noticed that there are many factors limiting the possibilities of detailed data analysis, starting from ethical problems enabling the collection of intrauterine material, through numerous factors influencing the quality of research—including environmental factors (e.g., maternal lifestyle, hygiene, diet, pollution, chemicals and stimulants) and technological factors, enabling proper identification of pathogens—up to research financing. Nevertheless, understanding the exact mechanisms of the relationship between intestinal microbiota and the functioning of the body is an extremely important aspect that requires further intensified research. Therefore, it seems reasonable to conduct research identifying the microbiota of the fetus and newborn with maximum protection of the identification process from interfering factors. In summary, the examination of the neonatal microbiota was performed immediately after birth, by cesarean section, from both full-term and premature pregnancies, proceeding without complications with the appropriate attitude of the pregnant woman adhering to the appropriate lifestyle and hygiene based on both maternal (e.g., placenta, throat swab and stool) and fetal—neonatal (e.g., gastric fluid, rectal swab and skin swab) material.

## 4. Conclusions

In the present review, we showed a relationship between the proper maternal microbiota and the normal functioning of the fetal and neonatal immune system. The state of eubiosis with an adequate amount and diversity of the microbiota is the basis for preventing the development of immune and allergic diseases. The way it is shaped, resulting from the health-promoting behavior of pregnant women, the rational conduct of the medical staff and the proper performance of the diagnostic and therapeutic process, is necessary to maintain the health of the mother and the child. Therefore, an appropriate lifestyle, adequate physical activity, proper diet, rational antibiotic therapy and the way of pregnancy completion are indispensable in the prevention of the above-mentioned conditions. Regarding the intestinal microbiota of the newborn in relation to the genera and phyla of bacteria that have a potentially protective effect, i.e., the phylum Firmicutes from the genus *Lactobacillus* and the phylum Bacteroidetes from the genus *Prevotella*, it is worth noting that the use of suitable probiotics and prebiotics seems to contribute to the protective effect. It is also extremely important to maintain an appropriate variety of bacterial phyla in order to maintain the state of eubiosis.

## Figures and Tables

**Table 1:** The table shows the dominant intestinal microbiota in newborns born vaginally shown by individual authors presented in this review.

Author	Kalbermattera et al. [145]	Méndez et al. [148]	Yao et al. [147]	Messa et al. [149]	Coelho et al. [150]
Bacterium	*Lactobacillus*	*Lactobacillus*	*Lactobacillus*	*Lactobacillus*	*Lactobacillus*
	*Bacteroides*	*Bacteroides*	*Enterococcaceae*	*Prevotella*	*Staphylococcus*
	*Bifidobacterium*	*Bifidobacterium*	*Streptococcaceae*	*Sneathia* spp.	
		*Lachnospiraceae*	*Clostridiaceae*		
			*Bacteroides*		
			*Bifidobacterium*		

**Table 2:** The table presents the dominant intestinal microbiota in newborns born by cesarean section shown by individual authors presented in this review.

Author	Rutavisire et al. [119]	Gabinelli et al. [147]	Méndez et al. [141]	Rachid et al. [148]	Eun et al. [149]
Bacterium	*Staphylococcus*	*Enterococcaceae*	*Enterococcaceae*	*Enterococcaceae*	Bacteroidetes
	*Streptococcus*	*Enterobacteriaceae*	*Enterobacteriaceae*	*Enterobacteriaceae*	Firmicutes *
	*Clostridium*	*Staphylococcus*	*Staphylococcus*	*Staphylococcus*	
		*Propionibacterium*	*Propionibacterium*	*Propionibacterium*	

* The author showed a greater diversity of a given type of bacteria.
